# Associations between Variants in *BDNF*/*BDNFOS* Gene and Lumbar Disc Herniation Risk among Han Chinese People

**DOI:** 10.1038/s41598-018-31146-6

**Published:** 2018-08-24

**Authors:** Yong Zhu, Haiyu Jia, Jiabin Li, Shaodong Ren, Zhi Huang, Feng Li, Wenhua Xing, Shunan Li, Xuejun Yang

**Affiliations:** 1 The Second Affiliated Hospital of Inner Mongolia Medical University, Hohhot, 010030 China; 2 The Affiliated Hospital of Inner Mongolia Medical University, Hohhot, 010000 China; 3 Inner Mongolia Medical University, Hohhot, 010050 China; 4 The Hohhot First Hospital, Hohhot, 010020 China

## Abstract

Lumbar disc herniation (LDH) is a low back pain disorder and associated with several single nucleotide polymorphisms (SNPs). However, the role of brain-derived neurotrophic factor (*BDNF*) and *BDNFOS* gene in LDH susceptibility remains unknown. To examine whether the variants contribute to LDH, 7 SNPs were genotyped in 380 patients and 692 healthy controls among Han Chinese population. Multiple genetic models, stratification by age/gender and haploview analysis was used by calculating odds ratio (OR) and 95% confidence intervals (CIs). Rs11030064 in *BDNFOS* gene was associated with modified susceptibility for LDH at age ≤50 years but three loci (rs6265, rs11030104 and rs10767664) of *BDNF* gene increased LDH risk at age >50 years. Further, rs11030096 polymorphism in *BDNFOS* gene was associated with LDH the increased susceptibility of LDH in females. Haplotype analysis shown that haplotype “GCC” in the block (rs988712, rs7481311, and rs11030064) increased LDH risk (OR = 1.49, 95% CI = 1.06–2.10, *p* = 0.022) at age ≤50 years. However, there was no significant association between *BDNF*/*BDNFOS* gene and LDH risk in the overall before stratified analysis. For the first time, our results provide evidence on polymorphism of *BDNF* / *BDNFOS* gene associated with LDH risk in Chinese Han population.

## Introduction

Lumbar disc herniation (LDH) is one of the more common spinal diseases caused by the degeneration and the displacement of nucleus pulposus or annulus fibrosis beyond the intervertebral disc space^1^. LDH is characterized with low back and leg pain resulting from the degenerated lumbar disc compressing the spinal nerve root. It is currently believed that some inflammatory mediators contribute to the radicular pain besides mechanical deformation. Recently, studies have proven that Th17 cells and IL-17 was infiltrated and expressed in intervertebral disc tissues and may contribute a lot to the local inflammation and radicular pain^2^. In addition, the accumulation of lymphocytes in local disc tissues is following exposure to autologous nucleus pulposus or injury to the annulus fibrosus^3^. Inflammatory cells from the intervertebral disc tissues can secrete many pro-inflammatory mediators and regulatory cytokines, such as TNF-α, IL-1β^4^, nerve growth factor^5^, and vascular endothelial growth factor^6^. Numerous studies of its etiology and pathogenesis indicated that LDH is a complicated and multifactorial spine disease affected by various factors, including gender, age, height, smoking habits, physical activity, occupation, vibration trauma, and so on^7,8^. Despite the specific factors influence susceptibility of symptomatic LDH is still unknown, genetic factors as an important role in the pathogenesis of LDH is gradually acknowledged^9^. Recent molecular epidemiological studies have pointed to the potential and significant role of polymorphisms in genes associated with various diseases such as LDH^10–12^.

Brain-derived neurotrophic factor (BDNF) known as ANON2 or BULN2 is a type of neurotrophin which include nerve growth factor, neurotrophin 3, and neurotrophin 4^13^. BDNF is located on 11p14.1, spanning approximately 70 kb and containing 11 exons. BDNF, widely distributed in the central nervous system, is a prominent role in the survival, differentiation, growth and development of neurons^14^. BDNFOS is also mapped in chromosome 11p14.1 and encoded by the genomic locus encoding BDNF. BDNFOS a primate-specific lncRNA that is natural antisense transcript positioned downstream of BDNF in reverse orientation, so it also is known as BDNF antisense RNA (BDNFAS) gene. Previous studies report that BDNFOS has a potential post-transcriptional regulation of BDNF through the formation of double-stranded duplexes^15^. Baker-Herman *et al*. found that the increase in BDNF was necessary and sufficient for spinal respiratory plasticity of spinal injury^16^. BDNF is known as a crucial neuromodulator involved in nociceptive hypersensitivity in the central nervous system and BDNF levels are modified in some persistent pain states as well as in inflammation^17,18^. Data accumulated in recent years suggest that *BDNF* gene has been regarded as a significant contributor to the spinal cord injury and spondyloarthritis^19,20^. Moreover, previous animal studies showed that BDNF also functions in chondrocyte and osteoblast, participates in cartilage development, ossification, and osteogenesis, and plays an important role in bone growth and development, remodeling and regeneration^21,22^. Consequently, studies concerning the possible association of *BDNFOS* and *BDNF* gene with LDH may be particularly interesting for their potential biological significance.

Overwhelming evidence indicates that BDNFOS and BDNF polymorphisms was associated with various spinal disease, such as spinal cord injury and related skeletal disease, including osteoporosis and osteoblast differentiation^23,24^. To the best of our knowledge, there are no previous studies have investigated the association of risk of LDH and *BDNFOS* and *BDNF* polymorphisms. Therefore, a case-control study was carried out to evaluate the possible association of *BDNFOS* and *BDNF* gene polymorphisms at allele, genotype, and haplotype interface with development of LDH among Chinese Han population.

## Results

### Characteristics of patients and controls

In this case-control study, we collected and analyzed 380 cases of LDH (228 males and 152 females) and 692 healthy controls (390 males and 302 females). The mean ages of the patients and the controls were 50.4 ± 12.3 and 48.2 ± 10.4 years, respectively. There were no statistically significant differences (*p* = 0.248) on the gender distribution between the case and control groups. However, the result revealed the age distribution was statistically significant differences (*p* < 0.001), suggesting that age may have an effect on the etiology of LDH.

The basic information of the SNPs regarding gene, SNP ID, chromosomal position, role, minor allele frequency (MAF) of cases and controls, Hardy-Weinberg equilibrium (HWE) test results and call rate, were shown in Table 1. Seven SNPs in the *BDNFOS* and *BDNF* gene were successfully genotyped for further analysis, and the call rate of SNPs was above 98.51% in case and controls. The genotype distribution of all SNPs in control subjects was in accordance with HWE (*p* > 0.05).

**Table 1:** Basic Information about *BDNFOS* and *BDNF* candidate SNPs in this study.

Gene	SNP ID	Location	Position	Role	Alleles		Frequency (MAF)		*p*-value for HWE	Call rate (%)
					A (minor)	B (major)	Cases	Controls		
BDNFOS	rs988712	11p14.1	27563382	Intron	T	G	0.136	0.137	0.256	98.51%
BDNFOS	rs7481311	11p14.1	27583129	Intron	T	C	0.299	0.292	0.927	99.91%
BDNFOS	rs11030064	11p14.1	27618016	Intron	T	C	0.392	0.413	0.389	99.81%
BDNFOS	rs11030096	11p14.1	27665543	Intron	C	T	0.330	0.317	0.291	99.35%
BDNF	rs6265	11p14.1	27679916	Coding exon	T	C	0.454	0.446	0.442	99.81%
BDNF	rs11030104	11p14.1	27684517	Intron	G	A	0.454	0.443	0.440	99.72%
BDNF	rs10767664	11p14.1	27725986	Intron	T	A	0.453	0.436	0.486	99.81%

SNP = single nucleotide polymorphism; MAF = minor allele frequency; HWE = Hardy-Weinberg equilibrium. p values were calculated with Pearson’s χ^2^ tests; p < 0.05 indicates statistical significance.

### The association between *BDNFOS* and *BDNF* gene and the risk of LDH

Genotype and allele frequencies of the SNPs among the LDH patients and control subjects were shown in Table 2. However, we had not found that any SNPs were significantly associated with LDH risk in the *BDNFOS* and *BDNF* gene at a 5% level (Table 2). Further, multiple inheritance models (dominant, recessive and additive models) were applied for analyzing the association by unconditional logistic regression analysis adjusted for age and gender, and there were also no statistically significant differences in patients and controls (*p* > 0.05) (As shown in Supplement Table 1).

**Table 2:** The genotype and allele frequencies of *BDNFOS* and *BDNF* in lumbar disc herniation patients and controls.

SNP ID	Genotype						Allele					
		Case	Control	OR (95% CI)	χ^2^	*p*		Case	Control	OR (95% CI)	χ^2^	*p*
rs988712	TT	6	16	0.69 (0.26–1.80)	0.932	0.627	T	103	185	0.994 (0.767–1.288)	0.002	0.962
	TG	91	153	1.07 (0.79–1.44)			G	655	1169	1		
	GG	282	508	1.00								
rs7481311	TT	34	58	1.07 (0.68–1.71)	0.114	0.944	T	227	404	1.031 (0.849–1.252)	0.095	0.758
	TC	159	288	1.01 (0.78–1.32)			C	533	978	1		
	CC	187	345	1.00								
rs11030064	TT	57	112	0.84 (0.57–1.23)	1.000	0.607	T	298	570	0.917 (0.765–1.098)	0.891	0.345
	TC	184	346	0.86 (0.65–1.13)			C	462	810	1		
	CC	139	232	1.00								
rs11030096	CC	44	75	1.12 (0.74–1.70)	0.367	0.832	C	250	435	1.060 (0.877–1.281)	0.364	0.546
	CT	162	285	1.08 (0.82–1.41)			T	508	937	1		
	TT	173	326	1.00								
rs6265	TT	75	142	1.03 (0.71–1.48)	1.145	0.564	T	345	615	1.034 (0.866–1.235)	0.136	0.712
	TC	195	331	1.13 (0.85–1.52)			C	415	765	1		
	CC	110	217	1.00								
rs11030104	GG	74	141	1.04 (0.72–1.49)	1.560	0.458	G	344	612	1.043 (0.873–1.246)	0.212	0.645
	GA	196	330	1.16 (0.87–1.56)			A	414	768			
	AA	109	219	1.00								
rs10767664	TT	75	136	1.10 (0.76–1.58)	1.349	0.509	T	344	602	1.069 (0.894–1.277)	0.534	0.465
	TA	194	330	1.16 (0.87–1.55)			A	416	778	1		
	AA	111	224	1.00								

OR = odds ratio; 95% CI = 95% confidence interval. *p* values were calculated with Pearson’s χ^2^ tests. *p* < 0.05 indicates statistical significance.

### Stratification analysis by age and gender

There were significant associations between the four SNPs and the risk of LDH in stratified analysis by age adjusted for age and gender, as displayed in Table 3. Rs11030064 in *BDNFOS* gene was observed to be associated with the reduced the susceptibility of LDH at age ≤50 years in codominant model (TC vs. CC, OR = 0.66, 95% CI = 0.45–0.98, *p* = 0.034), in dominant model (TC/TT vs. CC, OR = 0.65, 95% CI = 0.45–0.93, *p* = 0.010) and in log-additive model (OR = 0.74, 95% CI = 0.57–0.96, *p* = 0.023). Conversely, three loci (rs6265, rs11030104 and rs10767664) of *BDNF* gene increased LDH risk at age >50 years were discovered based on the results of the dominant model (rs6265, TC/TT vs. CC, OR = 1.50, 95% CI = 1.00–2.27, *p* = 0.035; rs11030104, GA/GG vs. AA, OR = 1.53, 95% CI = 1.01–2.31, *p* = 0.029; and rs10767664, AT/TT vs. AA, OR = 1.58, 95% CI = 1.05–2.38, *p* = 0.020) and the log-additive model(rs10767664, OR = 1.32, 95% CI = 1.01–1.73, *p* = 0.039).

**Table 3:** The relationship of *BDNFOS* and *BDNF* genetic polymorphisms with lumbar disc herniation according to the age stratification adjusted for age and gender.

SNP ID	Model	Genotype	>50 years				≤50 years			
			Control	case	OR (95% CI)	p	Control	case	OR (95% CI)	p
rs11030064	Codominant	C/C	98 (34.6%)	61 (30.1%)	1.00	0.68	134 (32.9%)	78 (44.1%)	1.00	**0**.**034**
		T/C	139 (49.1%)	107 (52.7%)	1.19 (0.78–1.80)		207 (50.9%)	77 (43.5%)	**0**.**66** (**0**.**45–0**.**98**)	
		T/T	46 (16.2%)	35 (17.2%)	1.20 (0.69–2.08)		66 (16.2%)	22 (12.4%)	0.59 (0.34–1.03)	
	Dominant	C/C	98 (34.6%)	61 (30.1%)	1.00	0.38	134 (32.9%)	78 (44.1%)	1.00	**0**.**010**
		T/C-T/T	185 (65.4%)	142 (70%)	1.19 (0.80–1.77)		273 (67.1%)	99 (55.9%)	**0**.**65** (**0**.**45–0**.**93**)	
	Recessive	C/C-T/C	237 (83.8%)	168 (82.8%)	1.00	0.76	341 (83.8%)	155 (87.6%)	1.00	0.25
		T/T	46 (16.2%)	35 (17.2%)	1.08 (0.66–1.76)		66 (16.2%)	22 (12.4%)	0.74 (0.44–1.24)	
	Log-additive	—	—	—	1.11 (0.85–1.45)	0.45	—	—	**0**.**74** (**0**.**57–0**.**96**)	**0**.**023**
rs6265	Codominant	C/C	93 (33%)	49 (24.1%)	1.00	0.14	124 (30.4%)	61 (34.5%)	1.00	0.46
		T/C	137 (48.6%)	111 (54.7%)	1.51 (0.98–2.33)		194 (47.5%)	84 (47.5%)	0.88 (0.59–1.32)	
		T/T	52 (18.4%)	43 (21.2%)	1.48 (0.87–2.55)		90 (22.1%)	32 (18.1%)	0.72 (0.43–1.21)	
	Dominant	C/C	93 (33%)	49 (24.1%)	1.00	**0**.**035**	124 (30.4%)	61 (34.5%)	1.00	0.34
		T/C-T/T	189 (67%)	154 (75.9%)	**1**.**50** (**1**.**00–2**.**27**)		284 (69.6%)	116 (65.5%)	0.83 (0.57–1.21)	
	Recessive	C/C-T/C	230 (81.6%)	160 (78.8%)	1.00	0.59	318 (77.9%)	145 (81.9%)	1.00	0.28
		T/T	52 (18.4%)	43 (21.2%)	1.14 (0.72–1.80)		90 (22.1%)	32 (18.1%)	0.78 (0.50–1.23)	
	Log-additive	—	—	—	1.24 (0.95–1.62)	0.11	—	—	0.85 (0.67–1.10)	0.22
rs11030104	Codominant	A/A	93 (32.9%)	48 (23.8%)	1.00	0.13	126 (31%)	61 (34.5%)	1.00	0.43
		G/A	139 (49.1%)	111 (55%)	1.52 (0.99–2.35)		191 (46.9%)	85 (48%)	0.92 (0.62–1.37)	
		G/G	51 (18%)	43 (21.3%)	1.54 (0.89–2.65)		90 (22.1%)	31 (17.5%)	0.72 (0.43–1.20)	
	Dominant	A/A	93 (32.9%)	48 (23.8%)	1.00	**0**.**029**	126 (31%)	61 (34.5%)	1.00	0.41
		G/A-G/G	190 (67.1%)	154 (76.2%)	**1**.**53** (**1**.**01–2**.**31**)		281 (69%)	116 (65.5%)	0.85 (0.59–1.25)	
	Recessive	A/A-G/A	232 (82%)	159 (78.7%)	1.00	0.5	317 (77.9%)	146 (82.5%)	1.00	0.21
		G/G	51 (18%)	43 (21.3%)	1.17 (0.74–1.86)		90 (22.1%)	31 (17.5%)	0.75 (0.48–1.19)	
	Log-additive	—	—	—	1.26 (0.97–1.65)	0.087	—	—	0.86 (0.67–1.10)	0.22
rs10767664	Codominant	A/A	96 (33.9%)	49 (24.1%)	1.00	0.056	128 (31.4%)	62 (35%)	1.00	0.48
		A/T	139 (49.1%)	110 (54.2%)	**1**.**54** (**1**.**00–2**.**37**)		191 (46.9%)	84 (47.5%)	0.91 (0.61–1.35)	
		T/T	48 (17%)	44 (21.7%)	1.70 (0.99–2.92)		88 (21.6%)	31 (17.5%)	0.73 (0.44–1.22)	
	Dominant	A/A	96 (33.9%)	49 (24.1%)	1.00	**0**.**020**	128 (31.4%)	62 (35%)	1.00	0.41
		A/T-T/T	187 (66.1%)	154 (75.9%)	**1**.**58** (**1**.**05–2**.**38**)		279 (68.5%)	115 (65%)	0.85 (0.59–1.24)	
	Recessive	A/A-A/T	235 (83%)	159 (78.3%)	1.00	0.29	319 (78.4%)	146 (82.5%)	1.00	0.27
		T/T	48 (17%)	44 (21.7%)	1.29 (0.81–2.04)		88 (21.6%)	31 (17.5%)	0.77 (0.49–1.22)	
	Log-additive	—	—	—	**1**.**32** (**1**.**01–1**.**73**)	**0**.**039**	—	—	0.86 (0.67–1.11)	0.24

OR = odds ratio; 95% CI = 95% confidence interval. *p* values were calculated with Pearson’s χ^2^ tests; *p* < 0.05 indicates statistical significance.

Furthermore, we conducted another stratified analysis of gender adjusted for age and found only rs11030096 polymorphism in *BDNFOS* gene was associated with LDH, as shown in Table 4. An increased risk of LDH for CC genotype was found among the female subgroup in the recessive model (TT/TC vs. CC, OR = 1.98, 95% CI = 1.05–3.73, *p* = 0.036), but not between the male subgroup in any genetic model.

**Table 4:** Association of rs110300 96 and lumbar disc herniation risk stratified by gender adjusted for age.

Model	Genotype	Male				Female			
		Control	case	OR (95% CI)	*p*	Control	case	OR (95% CI)	*p*
Genotype	T/T	187 (48.5%)	95 (41.9%)	1.00	0.065	139 (46.3%)	78 (51.3%)	1.00	0.034
	T/C	148 (38.3%)	109 (48%)	**1**.**44** (**1**.**02–2**.**05**)		137 (45.7%)	53 (34.9%)	0.72 (0.47–1.10)	
	C/C	51 (13.2%)	23 (10.1%)	0.89 (0.51–1.54)		24 (8%)	21 (13.8%)	1.70 (0.88–3.29)	
Dominant	T/T	187 (48.5%)	95 (41.9%)	1.00	0.12	139 (46.3%)	78 (51.3%)	1.00	0.45
	T/C-C/C	199 (51.5%)	132 (58.1%)	1.30 (0.93–1.81)		161 (53.7%)	74 (48.7%)	0.86 (0.58–1.27)	
Recessive	T/T-T/C	335 (86.8%)	204 (89.9%)	1.00	0.26	276 (92%)	131 (86.2%)	1.00	**0**.**036**
	C/C	51 (13.2%)	23 (10.1%)	0.74 (0.44–1.25)		24 (8%)	21 (13.8%)	**1**.**98** (**1**.**05–3**.**73**)	
Log-additive	—	—	—	1.08 (0.85–1.37)	0.55	—	—	1.06 (0.79–1.43)	0.7

OR = odds ratio; 95% CI = 95% confidence interval. *p* values were calculated with Pearson’s χ^2^ tests adjusted by age; *p* < 0.05 indicates statistical significance.

### Haplotype analyses

**Figure 1:**
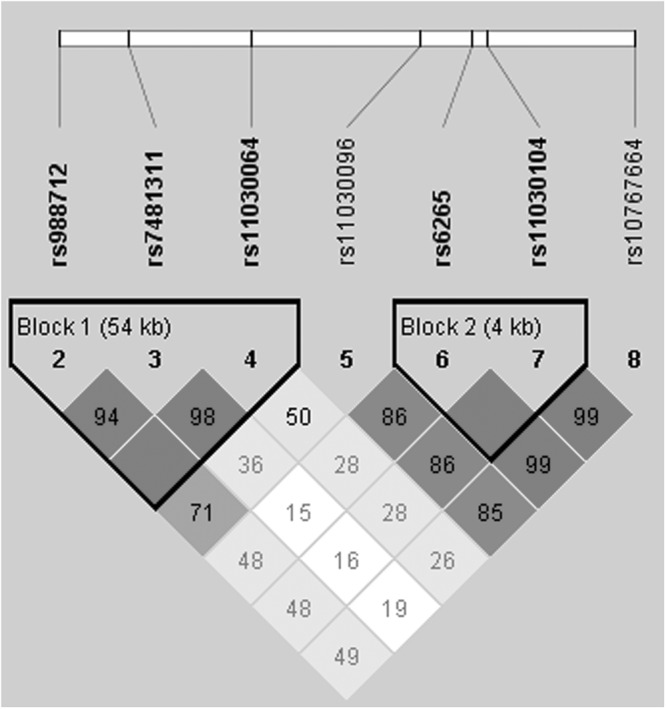
Haplotype block map for seven SNPs in *BDNF/BDNFOS* Gene at age <50 years.

The LD and haplotype analyses of the SNPs in the case and control samples were further studied. Among the subpopulation (age ≤50 years), seven SNPs were found to exist in two LD blocks (Block 1: rs988712, rs7481311and rs11030064; Block 2: rs6265 and rs11030104) in *BDNFOS* and *BDNF* gene (Fig. 1). The distributions of different haplotypes of *BDNFOS* and *BDNF* gene in both the patients of LDH and controls are presented in Table 5. The results showed that the GCT haplotype was lower in LDH patients than controls (*p* = 0.017), but the GCC haplotype was slightly higher (*p* = 0.058). Furthermore, haplotype GCC in Block 1 was found to significantly increase the risk of LDH under unconditional logistic regression analysis adjusted for age and gender (OR = 1.49, 95% CI = 1.06–2.10, *p* = 0.022) (Table 5). However, no relation was found between the other haplotypes and LDH risk.

**Table 5:** Haplotype frequencies and their associations with lumbar disc herniation risk at age ≤50 years.

	Haplotype	Frequency		χ^2^	*P* value	Crude analysis		Adjusted by age and gender	
		Case	Control			OR (95% CI)	*p*	OR (95% CI)	*p*
Block 1	GCT	0.341	0.415	5.661	0.0173	1.00	—	1.00	—
	GTC	0.298	0.275	0.648	0.4207	1.34 (0.98–1.85)	0.071	1.34 (0.97–1.85)	0.072
	GCC	0.221	0.174	3.593	0.058	**1**.**54** (**1**.**10**–2.17)	**0**.**013**	**1**.**49** (**1**.**06**–2.10)	**0**.**022**
	TCC	0.130	0.132	0.013	0.9096	1.20 (0.80–1.82)	0.38	1.16 (0.77–1.76)	0.48
Block 2	CA	0.582	0.543	1.532	0.2158	1.00	—	1.00	—
	TG	0.415	0.456	1.66	0.1976	0.85 (0.67–1.09)	0.21	0.86 (0.67–1.10)	0.22

Block 1 comprised of the three closely linked SNPs rs988712, rs7481311, and rs11030064. Block 2 comprised of the two closely linked SNPs rs6265 and rs11030104. OR = odds ratio; 95% CI = 95% confidence interval. *p* values were calculated with Pearson’s χ^2^ tests; *p* < 0.05 indicates statistical significance.

## Discussion

Lumbar disc herniation, one of the most typical lumbar diseases, is determined by various genetic and environmental interactions. It is widely acknowledged that hereditary factors contribute prominently to the risk of LDH and a large amount of genes and SNPs have been identified to be associated with LDH^25^. However, the contribution of the in SNPs *BDNS*/*BDNSOF* gene to LDH is still unclear. Take into account these, in this case-control study, allele, genotype and haplotype frequencies of seven SNPs in the *BDNS*/*BDNSOF* gene between LDH patients and healthy controls were compared and stratification analyses by age or gender were conducted. There was not any association was found between polymorphisms and LDH risk in the overall. Our study found three of the seven SNPs (rs6265, rs11030104 and rs10767664) possibly contributed to the susceptibility of LDH at age >50 years, and rs11030064 and GCC haplotype in the block (rs988712, rs7481311, and rs11030064) were related with the risk of LDH at age ≤50 years. In addition, we also found that rs11030096 polymorphism conferred the increased susceptibility to LDH in the female population. To our knowledge, this is the first study that evaluated and showed an association of *BDNS* and *BDNSOF* genetic variants with risk of developing LDH in China Han population.

LDH, a degenerative disease related to narrowing of the spinal canal or intervertebral foramina, can lead to spinal pain syndromes and radicular symptoms caused by the generation of nerve root ischemia^26^. In addition, various inflammatory related factors play a role in inducing lumbar disc degeneration and nervous radical pain, and further accelerating inflammation and intervertebral disc generation; thus, this vicious circle worsens lumbar disc degeneration and pain^27,28^. BDNF, a neurotrophic protein, is not only related to neuroprotective but also nociceptive hypersensitivity in the central nervous system. There is compelling evidence that BDNF levels are regulated in some persistent pain states as well as in inflammation^29^. The function of BDNF in the central nervous system has been studied in detail but a role of neurotrophins as important factors in inflammation has an open research question. BDNF is reported to be involved in inflammatory reactions, and its production is increased in response to pro-inflammatory cytokines^30^. Some studies are manifested that high BDNF mRNA expression levels have been detected in the synovial fluid cells of osteoarthritis, rheumatoid arthritis, and spondyloarthritis patients^31^. Spondyloarthritis is the most common pathological change in the spine. Depending on the clinical symptoms there are two basic pathological syndromes: spondylosis related mainly to the intervertebral disc and bone structures of vertebras and myelopathy including spinal cord injury caused by compression^32,33^. Therefore, we propose a reasonable hypothesis that the pathogenesis of LDH is associated with BDNF.

BDNF is a crucial factorin the cell cycle, neurite outgrowth, and synaptic plasticity and has been linked to many human brain disorders^34^. Sequence variations in the *BDNF* gene may lead to variations in gene expression, further to affect the function of the protein. Several single-nucleotide polymorphisms (SNPs) in the *BDNF* gene have been identified, including rs6265, rs11030104 and rs10767664 polymorphisms which are located in the coding exon, promoter and intron region, respectively. Rs6265 (Val66Met), a common substitution of G to A, was identified in the 5′pro-BDNF sequence which leads to a change of Valine (Val) to Methionine (Met) at codon 66. This substitution seemed to be of functional significance to affect intracellular trafficking, packaging of proBDNF, and activity dependent secretion of BDNF^35^. In the present case-control study, our result shows that rs6265 and two intronic SNPs, rs11030104 and rs10767664 in *BDNF* gene exhibited an increased risk of LDH in the dominant model at age >50 years, suggesting that these *BDNF* variants are likely to be susceptibility markers for LDH. We speculate it seems like a plausible explanation that age-related degenerative changes occurring within the lumbar intervertebral disc also be affected by the transcription efficiency and function of the *BDNF* gene. However, the mechanisms still need more functional studies to testify. Moreover, we could not exclude the possibility that the deficiency of association in a certain group might be a consequence of the limited sample size.

BDNFOS, positioned downstream of BDNF, play an important role in tissue-specific regulation of BDNF. It has been reported that inhibition of BDNF-AS upregulates BDNF mRNA, which subsequently increases protein levels and stimulates neuronal outgrowth and differentiation^36,37^. However, studies based on this *BDNFOS* gene are very few. In our result, rs11030064 in *BDNFOS* gene was found by the association with decreased LDH risk in codominant, dominant and log-additive models at age ≤50 years. In addition, we also found rs11030096 of *BDNFOS* gene in the recessive model (CC vs. TT-TC) has increased 1.98-fold risk of LDH in females compared to males. This result demonstrated that rs11030096 of *BDNFOS* are associated with susceptibility to LDH in Chinese Han population and also the risk association of the polymorphisms is gender dependent. However, these results mean that larger sample sets are needed to affirm and *BDNF/BDNFOS* gene participated in lumbar disc degeneration is desired to investigate in future studies.

Several limitations of this study should not be ignored. Firstly, the subjects of investigation were enrolled from the identical hospital and therefore selection bias is could not rule out. However, all observed genotype frequencies in controls were in agreement with HWE, which may reduce the bias to some degree. Secondly, the number of cases in our study was not large we cannot preclude false-negative results. So, larger sample size and further confirmation in other ethnic populations are needed for further verification. Thirdly, some potential confounding factors such as occupational exposures, and physical activity were not included in our analysis and should be assessed in the future. Despite the limitations mentioned above, the results of our present study provided scientific evidence about *BDNS/BDNSOF* gene with LDH in the future studies.

In summary, our present study provided evidence that the variants of *BNDF/BDNFOS* gene had a significant effect on the risk of LDH in the Chinese Han population especially more than 50 years old, which has not previously been reported. Although a larger sample size, more diverse populations should be conducted to confirm and extend our findings, we believe that the *BNDF/BDNFOS* gene is a new insight into the LDH and the functional role of *BNDF/BDNFOS* gene in LDH is necessary to study.

## Materials and Methods

### Study participants

Using a case-control design, 380 patients with LDH and 692 controls were enrolled. All patients were recruited from the Second Affliated Hospital of Inner Mongolia Medical University and The Hohhot First Hospital. The inclusion criteria for patients were: patients who had typical clinical symptoms and physical signs; and patients with LDH confirmed by image examinations including computed radiography, computed tomography, and/or magnetic resonance imaging (MRI). LDH symptoms were those described as follows: (1) lower back pain, (2) pain in the inferior lumbar part of the spine and regional typical sciatica; (3) difficulty in straight-leg raising test and augmentation test; (4) the limited lumbar flexion range^38^. Lumbar spine MRI confirmed the patients with LDH according to the Pfrrmann grading system^39^. Patients with complicated blood diseases, autoimmune diseases, tumors, trauma, rheumatoid arthritis, and related lumbar spine disease containing lumbar spinal stenosis, spinal congenital dysplasia, intraspinal tumor, and spondylolisthesis were excluded from this study. The controls were healthy volunteers from the medical examination during the same period and not scanned by MRI and no history of sciatica and low back pain. Inclusion criteria of the control group were: (1) individuals had no medical and family history of lumbocrural pain; (2) individuals without spinal instability from trauma, scoliosis, spondylolisthesis, osteoarthritis, rheumatism and rheumatoid arthritis; (3) individuals without infections and any history of tumors. All studied individuals were Chinese Han subjects tracing back at least three generations.

### Data collection

This study was approved by the ethics committee of the Second Affliated Hospital of Inner Mongolia Medical University and The Hohhot First Hospital, and conformed to the ethical principles for medical research involving humans of the World Medical Association Declaration of Helsinki. All participants signed informed consent forms before participating in this study. Individual demographics information was collected with a standard questionnaire conducted by well-trained interviewers. Subsequently, about 5 mL venous blood sample from each participant was collected into tubes containing ethylenediamine tetraacetic acid (EDTA) for anticoagulation. Genomic DNA was extracted from peripheral blood samples using a Whole Blood Genomic DNA Extraction Kit (Tiangen Biotech, Beijing, China) according to the manufacturer’s instructions. The purity and concentration of the DNA samples were evaluated with the NanoDrop 2000C (Thermo Scientifc, Waltham, Massachusetts, USA). The isolated DNA was stored at −80 °C until analysis.

### SNP genotyping

Seven candidate SNPs in the *BDNFOS* and *BDNF* gene were selected with the minor allele frequency (MAF) >0.05 in Han Chinese from the 1000 Genome Projects. Sequenom MassARRAY assay (Sequenom, San Diego, CA, USA) was used for detection of *BDNFOS* and *BDNF* gene polymorphisms. The primers for amplification and single base extension were designed using the Sequenom MassARRAY Assay Design 3.0 Software (San Diego, California, USA). The data was collected and analyzed by Sequenom Typer 4.0 Software as previously described^40^. Genotyping was carried out by two laboratory personnel in a double-blinded fashion. In order to confirm the results, about 10% of the samples were randomly selected to repeat genotyping and the reproducibility was 100%.

### Data analyses

The Pearson’s χ^2^ test and independent sample Student’s t-test were applied to assess the differences in the distribution of demographic characteristics between cases and controls. Fisher’s exact tests for HWE were performed by comparing the observed and expected genotype frequencies to calculate the genotype frequencies among the controls. Pearson Chi-squared test or fisher exact test was used to compare the allelic and genotype frequencies of each SNP between patients with LDH and controls. Odds ratios (OR) and 95% confidence intervals (CI) were calculated to estimate the association between *BDNFOS*/*BDNF* gene and the risk of LDH using unconditional logistic regression analysis with or without adjustment for potential confounding. The wild-type allele was used as a reference. Multiple genetic model analyses (codominant, dominant, recessive and log-additive) were applied using SNPstats software (http://bioinfo.iconcologia.net/snpstats/start.htm) to assess the association between SNPs and LDH. Further, we calculated stratification factors using age (≤50 and >50 years) and gender (male and female) to adjust for possible cofounders. Finally, the pairwise linkage disequilibrium (LD), haplotype construction and genetic association of polymorphism loci were assessed using the Haploview software package (version 4.2) and the SHEsis software (http://analysis.bio-x.cn/myAnalysis.php). Statistical analyses were performed using SPSS software (version 21.0, IBM Corporation, Armonk, NY, USA). All *p* values of statistical tests were two-sided, and *p* ≤ 0.05 was considered as statistically significant.

## Electronic supplementary material

Supplementary Information

## Data Availability

All data generated or analyzed during this study are included in this manuscript.
