# Supplementary material for: Associations between Variants in BDNF/BDNFOS Gene and Lumbar Disc Herniation Risk among Han Chinese People
Source: Sci Rep. 2018 Aug 24;8:12782. doi: 10.1038/s41598-018-31146-6 (PMC6109184; doi:10.1038/s41598-018-31146-6)
Supplement: Supplementary file 1 — Supplementary Information [file 41598_2018_31146_MOESM1_ESM.pdf]

Associations between Variants in *BDNF / BDNFOS* Gene and Lumbar Disc Herniation Risk among Han Chinese People

Yong Zhu[1,*], Haiyu Jia[2,*], Jiabin Li[3], Shaodong Ren[3], Zhi Huang[1], Feng Li[1], Wenhua Xing[1], Shunan Li[4,#], Xuejun Yang[1,#]

[1.] The Second Affiliated Hospital of Inner Mongolia Medical University, Hohhot 010030, China

[2.] The Affiliated Hospital of Inner Mongolia Medical University, Hohhot 010000, China.

[3.] Inner Mongolia Medical University, Hohhot 010050, China

[4.] The Hohhot First Hospital, Hohhot 010020, China

[*]Co-first authors.

[#]Corresponding Author:

Shunan Li.

Tel/Fax: 13848136340

Email: 522641997@qq.com

Address: #150, South second ring, Yuquan District, Hohhot 010020, Inner Mongolia autonomous region, China

Xuejun Yang.

Tel/Fax: 15804712191

Email: xuejun__yang@163.com

Address: #1, Yingfang Road, Huimin District, Hohhot 010030, Inner Mongolia autonomous region, China

Supplement Table 1 Relationship between *BDNF/BNDFOS* gene polymorphisms and risk of LDH under multiple models of inheritance.

SNP ID	Model	Genotype	Control	case	Adjusted by age and gender	
					OR(95%CI)	p-value
rs988712	Dominant	G/G	508 (75%)	282 (74.4%)	1.00	0.81
		G/T-T/T	169 (25%)	97 (25.6%)	1.04 (0.77-1.39)	
	Recessive	G/G-G/T	661 (97.6%)	373 (98.4%)	1.00	0.41
		T/T	16 (2.4%)	6 (1.6%)	0.68 (0.26-1.76)	
	Log-additive	---	---	---	1.00 (0.77-1.29)	0.98
rs7481311	Dominant	C/C	345 (49.9%)	187 (49.2%)	1.00	0.87
		C/T-T/T	346 (50.1%)	193 (50.8%)	1.02 (0.79-1.31)	
	Recessive	C/C-C/T	633 (91.6%)	346 (91%)	1.00	0.77
		T/T	58 (8.4%)	34 (8.9%)	1.07 (0.68-1.67)	
	Log-additive	---	---	---	1.03 (0.84-1.25)	0.8
rs11030064	Dominant	C/C	232 (33.6%)	139 (36.6%)	1.00	0.24
		T/C-T/T	458 (66.4%)	241 (63.4%)	0.85 (0.65-1.11)	
	Recessive	C/C-T/C	578 (83.8%)	323 (85%)	1.00	0.62
		T/T	112 (16.2%)	57 (15%)	0.91 (0.65-1.30)	
	Log-additive	---	---	---	0.90 (0.75-1.09)	0.27
rs11030096	Dominant	T/T	326 (47.5%)	173 (45.6%)	1.00	0.53
		T/C-C/C	360 (52.5%)	206 (54.4%)	1.08 (0.84-1.40)	
	Recessive	T/T-T/C	611 (89.1%)	335 (88.4%)	1.00	0.71

		C/C	75 (10.9%)	44 (11.6%)	1.08 (0.72-1.61)	
	Log-additive	---	---	---	1.06 (0.88-1.28)	0.52
rs6265	Dominant	C/C	217 (31.4%)	110 (28.9%)	1.00	0.49
		T/C-T/T	473 (68.5%)	270 (71%)	1.10 (0.84-1.45)	
	Recessive	C/C-T/C	548 (79.4%)	305 (80.3%)	1.00	0.74
		T/T	142 (20.6%)	75 (19.7%)	0.95 (0.69-1.30)	
	Log-additive	---	---	---	1.02 (0.86-1.22)	0.79
rs11030104	Dominant	A/A	219 (31.7%)	109 (28.8%)	1.00	0.4
		G/A-G/G	471 (68.3%)	270 (71.2%)	1.12 (0.85-1.48)	
	Recessive	A/A-G/A	549 (79.6%)	305 (80.5%)	1.00	0.71
		G/G	141 (20.4%)	74 (19.5%)	0.94 (0.69-1.29)	
	Log-additive	---	---	---	1.03 (0.86-1.23)	0.74
rs10767664	Dominant	A/A	224 (32.5%)	111 (29.2%)	1.00	0.34
		A/T-T/T	466 (67.5%)	269 (70.8%)	1.14 (0.87-1.50)	
	Recessive	A/A-A/T	554 (80.3%)	305 (80.3%)	1.00	0.99
		T/T	136 (19.7%)	75 (19.7%)	1.00 (0.73-1.37)	
	Log-additive	---	---	---	1.06 (0.88-1.27)	0.54

OR = odds ratio; 95% CI = 95% confidence interval. *p* values were calculated with Pearson′s χ^2 tests; $p \leq 0.05$ indicates statistical significance.
